# Hepatocyte Growth Factor-Preconditioned Neural Progenitor Cells Attenuate Astrocyte Reactivity and Promote Neurite Outgrowth

**DOI:** 10.3389/fncel.2021.741681

**Published:** 2021-12-09

**Authors:** James Hong, Rachel Dragas, Mohammad Khazaei, Christopher S. Ahuja, Michael G. Fehlings

**Affiliations:** ^1^Department of Genetics and Development, Krembil Research Institute, University Health Network, Toronto, ON, Canada; ^2^Institute of Medical Science, University of Toronto, Toronto, ON, Canada; ^3^Spinal Program, University Health Network, Toronto Western Hospital, Toronto, ON, Canada

**Keywords:** central nervous system injury, regeneration, astrogliosis, glial scar, neural progenitor cell, hepatocyte growth factor

## Abstract

The astroglial scar is a defining hallmark of secondary pathology following central nervous system (CNS) injury that, despite its role in limiting tissue damage, presents a significant barrier to neuroregeneration. Neural progenitor cell (NPC) therapies for tissue repair and regeneration have demonstrated favorable outcomes, the effects of which are ascribed not only to direct cell replacement but trophic support. Cytokines and growth factors secreted by NPCs aid in modifying the inhibitory and cytotoxic post-injury microenvironment. In an effort to harness and enhance the reparative potential of NPC secretome, we utilized the multifunctional and pro-regenerative cytokine, hepatocyte growth factor (HGF), as a cellular preconditioning agent. We first demonstrated the capacity of HGF to promote NPC survival in the presence of oxidative stress. We then assessed the capacity of this modified conditioned media (CM) to attenuate astrocyte reactivity and promote neurite outgrowth *in vitro*. HGF pre-conditioned NPCs demonstrated significantly increased levels of tissue inhibitor of metalloproteinases-1 and reduced vascular endothelial growth factor compared to untreated NPCs. In reactive astrocytes, HGF-enhanced NPC-CM effectively reduced glial fibrillary acidic protein (GFAP) expression and chondroitin sulfate proteoglycan deposition to a greater extent than either treatment alone, and enhanced neurite outgrowth of co-cultured neurons. *in vivo*, this combinatorial treatment strategy might enable tactical modification of the post-injury inhibitory astroglial environment to one that is more conducive to regeneration and functional recovery. These findings have important translational implications for the optimization of current cell-based therapies for CNS injury.

## Introduction

Despite major advances in the medical management and pathophysiological understanding of the central nervous system (CNS), traumatic brain injury (TBI) and spinal cord injury (SCI) remain major causes of death and impairment worldwide, carrying significant personal and socioeconomic ramifications and relatively few treatment options available (Kraus, 1996; Hyder et al., 2007; Cripps et al., 2011; Krueger et al., 2013; Najem et al., 2018).

CNS injury is a biphasic and dynamic event characterized by the primary injury, or mechanical insult, followed by a cascade of cellular and molecular events constituting the secondary injury phase. The primary injury predominately results in extensive tissue necrosis and vascular disruption, with the secondary injury further exacerbating cellular apoptosis, vascular disruption, as well as oxidative stress, neuroinflammation, and reactive astrogliosis (Kraus, 1996; Najem et al., 2018). This cascade of injury is well characterized in animal models of TBI and SCI, and faithfully recapitulates many clinical aspects of these injuries (Hellewell et al., 2016; Forgione et al., 2017).

To elaborate, oxidative stress through the generation of reactive oxygen species has been shown to disrupt the neurovascular unit leading to blood-spinal cord-barrier disruption and infiltration of leukocytes (Lin et al., 2007). On the other hand, reactive astrogliosis is a dynamic and indispensable response mechanism to neurological injury that is largely characterized by distinct changes in astrocyte morphology and molecular expression. Following an injury to the CNS, astrocytes are activated at and around the injury site, resulting in hypertrophy, proliferation, and a compact network of gap and tight junctions that ultimately culminate in the glial scar (Bush et al., 1999; Faulkner et al., 2004; Sofroniew, 2005; Okada et al., 2006). Reactive astrocytes undergo molecular changes, most notably an increase in expression of cytoskeletal filaments like glial fibrillary acidic protein (GFAP) as well as increased deposition of growth-inhibitory molecules such as chondroitin sulfate proteoglycans (CSPGs; Mckeon et al., 1995, 1999; Fitch and Silver, 1997, 2008).

While literature has increasingly highlighted the reparative response of the glial scar in limiting inflammatory cell infiltration and restoring the blood-brain/spinal cord barrier (Faulkner et al., 2004; Silver and Miller, 2004; Sofroniew, 2005; Anderson et al., 2016), its physical and chemical composition presents a substantial barrier to regeneration and functional recovery. As such, a less convoluted scar concomitant with reduced CSPG deposition may foster improved regeneration and recovery following injury (Noble et al., 2002).

Stem cell therapies are a rising and clinically attractive treatment option for the repair and regeneration of the injured CNS (Assinck et al., 2017; Hachem et al., 2017). While numerous cell types have been investigated for use in TBI and SCI, neural progenitor cells (NPCs) are particularly advantageous given their migratory capacity and ability to differentiate into the neuronal and glial lineages of the CNS (Arvidsson et al., 2002; Takeuchi et al., 2007; Cossetti et al., 2012; Khazaei et al., 2017). While NPCs are widely known for their cell replacement capacity, they are also a substantial source of trophic support. NPCs secrete an array of beneficial cytokines, chemokines, growth factors, and extracellular matrix molecules (ECM) molecules that have been shown to aid in modifying the inhospitable post-injury environment to one more permissive for tissue repair and regeneration (Pluchino et al., 2009; Cossetti et al., 2012). As such, NPC conditioned media (CM) has been increasingly investigated as a singular or combinatorial therapy for CNS injury, demonstrating reduced tissue damage, enhanced regeneration at the injury site, and improvements in locomotor function (Liang et al., 2014; Cheng et al., 2017; Doeppner et al., 2017).

Given the multifactorial nature of CNS injury, a combinatorial treatment paradigm is likely necessary to achieve optimal therapeutic efficacy. Our lab, among others, has demonstrated enhanced behavioral recovery after SCI with combinatorial treatments utilizing NPCs alongside cytokines, growth factors, or CSPG-degrading enzymes (Behrstock et al., 2006; Tom et al., 2009; Karimi-Abdolrezaee et al., 2010). Retaining the cell replacement capacity of NPCs while strategically enhancing the reparative capacity of NPC-CM, may prove particularly advantageous in modifying the hostile and growth-inhibitory post-injury environment to one that is more conducive to regeneration and functional recovery. Hepatocyte growth factor (HGF) is a multifunctional cytokine that regulates development, motility, and morphogenesis in a number of cell types. HGF and its tyrosine kinase receptor, c-Met, are expressed in the CNS, and, following injury, stimulate a number of neuroprotective and regenerative effects through MAPK and PI3-kinase/Akt signaling pathways (Honda et al., 1995; Hamanoue et al., 1996; Shimamura et al., 2007). Moreover, while several growth factors have been investigated alone or as combinatorial agents following CNS injury, studies have attested to the distinct advantages conferred by HGF in supporting cell survival and neuroregeneration among other reparative processes relative to well-known neurotrophic factors such as brain-derived neurotrophic factor (BDNF), ciliary neurotrophic factor (CNTF), and glial-derived neurotrophic factor (GDNF; Shang et al., 2010; Wong et al., 2014).

Considering the multifaceted reparative and pro-regenerative potential of HGF following CNS injury, we assessed its utility in enhancing NPC secretory capacity to target inhibitory facets of astrocyte reactivity. Through a non-viral and cost-effective approach, we show that combinatorial treatment using HGF-preconditioning or concurrent administration of HGF alongside NPC-CM effectively attenuates astrocyte reactivity and promotes neurite outgrowth to a greater degree than either treatment alone. These effects may in turn be mediated by distinct changes in NPC secretory cytokine levels. This is the first study to assess the effect of HGF on NPC trophic capacity, and, taken together, our findings support the further mechanistic and preclinical investigation of HGF-enhanced NPCs as a novel and potentially translatable glial-modifying therapy for traumatic CNS injury.

## Materials and Methods

### MTT Cell Viability Assay

Fifty thousand rat spinal cord-derived NPCs were seeded into each well of a Matrigel-coated 96-well plate and subjected to oxidative stress-induced conditions *via* 500 μM of hydrogen peroxide treatment for 24 h alongside concurrent administration of HGF at different concentrations (10, 20, and 50 ng/ml); this oxidative stress model was based on a previously published protocol using the cell same type and derivation (Hachem et al., 2015). MTT reagent (Sigma-Aldrich) was then applied in accordance with the manufacturer’s instructions. Cells were incubated for 4 h at 37°C in light-sensitive conditions, 100 μl/well of DMSO was applied, and incubated at 37°C until cells were lysed and subsequent crystals were dissolved. Absorbance was then measured at 570 nm. Resultant readings were expressed as the total number of viable NPCs compared to hydrogen peroxide-treated NPC controls, with biological replicates of *n* = 3 per treatment group.

### Isolation and Expansion of Primary Rodent NPCs

**Figure 1:**
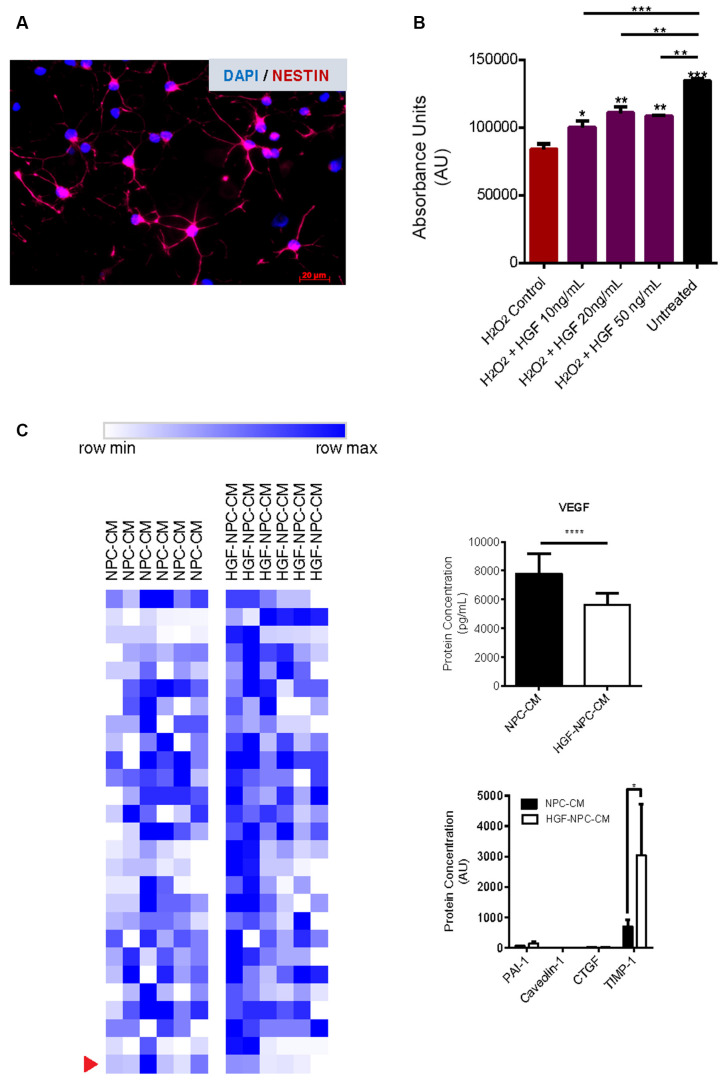
Hepatocyte growth factor (HGF)-preconditioned NSPCs demonstrate reduced levels of VEGF and increased TIMP-1 production. **(A)** Characterization of rat spinal cord-derived NSPCs grown in monolayer. Immunocytochemistry revealed cellular expression of the progenitor cell marker Nestin. Representative image taken at 20× using a Zeiss Axioplan 2 epifluorescence microscope. **(B)** Plated rat spinal cord-derived NSPCs were subjected to oxidative stress *via* hydrogen peroxide and treated for 24 h with recombinant human HGF at different concentrations. MTT reagent was applied in accordance with the manufacturer’s instructions and absorbance was measured at 570 nm. A reduction of approximately 40% was observed in the viable cell numbers of hydrogen peroxide-treated NSPCs relative to untreated cells. Concurrent administration of HGF protein to hydrogen peroxide-treated NSPCs demonstrated significantly increased cell viability relative to hydrogen peroxide-treated controls, with higher concentrations of HGF (20 ng/ml and 50 ng/ml) exhibiting the most notable effects. Data are expressed as mean ± SEM and presented as the total number of viable cells compared to hydrogen peroxide-treated NSPC controls. One-way ANOVA statistical analysis with Tukey’s *post hoc* test was performed; *p ≤ 0.05; **p ≤ 0.01; ***p ≤ 0.001; ****p ≤ 0.0001. **(C)** NSPCs were preconditioned using HGF protein at a concentration of 20 ng/ml based on measures of cell viability upon exposure to oxidative stress, as described previously. Secretory cytokine changes in HGF-preconditioned NSPCs were assessed using the Rat Cytokine Array/Chemokine Array 27 Plex (RD27) and Rat Vascular Injury P1-4-Plex kits, comparing the relative levels of 27 and four cytokines, respectively. Protein levels were normalized to untreated NPC-CM controls (*n* = 6). Heat mapping was used to depict relative protein concentrations (measured in pg/ml or AU) for each individual sample. Color gradient represents the range from row minimum to maximum. Of the proteins examined in the cytokine/chemokine and vascular arrays, VEGF and TIMP1 were the only proteins to reach significance (p-adjusted < 0.05), and their concentrations are shown as mean ± SEM.

All animal experiments were approved by the Animal Care Committee of the University Health Network (Project ID Code: #2212, Date of Approval: 17 May 2017) in compliance with the Canadian Council on Animal Care. NPCs were isolated from the central canal of the spinal cord in adult female Wistar rats, in accordance with a previously published protocol (Mothe and Tator, 2015). The spinal cord was extracted and washed in Dulbecco’s phosphate-buffered saline supplemented with 30% glucose and 1% penicillin/streptomycin (Sigma-Aldrich). The tissue from the spinal cord ependyma was harvested and enzymatically dissociated in a solution containing 0.01% papain and 0.01% DNase I for 1 h at 37°C. Next, the tissue was mechanically dissociated into a cell suspension and further centrifuged using a discontinuous density gradient to remove cell membrane fragments. Cells were resuspended in Neurobasal-A medium supplemented with B27 (Gibco/Invitrogen), GlutaMax (Gibco/Invitrogen), penicillin/streptomycin (Gibco/Invitrogen), 20 ng/ml EGF and FGF2 (Sigma Aldrich), 2 μg/ml heparin (Sigma-Aldrich), and hormone mix. Cells were cultured in uncoated Nunc culture flasks and incubated at 37°C, 5% CO_2_, and 90% humidity. The resultant neurospheres were passaged weekly *via* trituration-induced mechanical dissociation in the fresh medium described above. When grown as adherent cultures in monolayer, cells expressed the precursor marker Nestin (Figure 1A).

### Preparation, Collection, and Characterization of NPC Conditioned Media

At passage 4, neurospheres were collected, mechanically dissociated *via* trituration, and cultured at a density of 1,000 cells/μl in uncoated Nunc T25 culture flasks at 37°C, 5% CO_2_, and 90% humidity in the fresh medium as described above. Viable cell calculation was assessed using Trypan blue staining, and cell density was calculated *via* hemocytometer. Resultant neurosphere-containing media was collected 7 days later, centrifuged at 16,000 rpm for 5 min, and filtered using a syringe filter unit (0.44 μm; Millipore, Darmstadt, Germany) to remove any residual cells.

To generate HGF-preconditioned NPC-CM, 20 μg/μl of recombinant HGF protein (#100-39, PeproTech, Canada) was added to free-floating neurospheres grown in fresh growth medium (1,000 cells/μl). Human HGF is greatly conserved between mammals, with an amino acid sequence identity of 91% between humans and rats (Mizuno et al., 1994; Gherardi et al., 2003). As such, the protein was appropriate for the purposes of this study. A resultant concentration of 20 ng/ml was selected for preconditioning based on findings from the NPC oxidative stress assay mentioned previously. CM was collected 7 days later as formerly described.

Pre-conditioned and untreated NPC-CM were concentrated 25-fold using “Vivaspin 20” 3 kDa MWCO PES centrifugal concentrators (GE Healthcare Biosciences, Canada). Protein concentration was calculated using the microBCA assay (Thermo Scientific, Canada) according to the manufacturer’s instructions. 100 μl of sample (*n* = 6) protein was diluted at a 1:1 ratio with 1× PBS (200 μl total) and sent to Eve Technologies^1^ for high-throughput ELISA profiling using their 27-plex rat cytokine/chemokine array (RD27) and rat vascular injury panel P1. Proteins that contained interpolated or out-of-range signals were removed from the analyses. The proteins were then normalized using their calculated dilution factors and protein concentrations. The concentration of the proteins was either expressed in pg/ml derived from the company’s fluorescence standard curve (cytokine panel) or fluorescence intensity (vascular panel), and a one-way ANOVA with a Tukey’s *post hoc* test (alpha = 0.05) was used to compute the *p*-values. Protein concentration data from the cytokine/chemokine and vascular arrays are presented as row max and row min of individual mean protein concentrations (pg/ml). Heat mapping was done using the Morpheus software package from the Broad Institute^2^.

### Culture and Expansion of Primary Rat Astrocytes

Primary rat cortical-derived astrocytes (#N7745100; Gibco, Thermo Scientific, Canada), were thawed and expanded according to the manufacturer’s protocol. At passage 4, astrocytes were seeded in a monolayer on 10-cm plates for Western and Slot blots. Conversely, a 24-well plate with glass coverslips was used to grow astrocytes for immunocytochemistry (VWR International, Ontario). In both cases, plates and coverslips were coated with Matrigel (Thermo Scientific, Canada) and astrocytes were grown in a medium containing 89% Dulbecco’s Modified Eagle Medium/F12 containing no phenol red (DMEM; Gibco/Invitrogen), 10% fetal bovine serum (FBS), and 1% 100 U/ml penicillin/100 μg/ml streptomycin (Gibco/Invitrogen). Cells were incubated at 37°C, 5% CO_2_, and 90% humidity, with the medium changed every 3–4 days.

### Astrocyte Activation and Treatment

Astrocytes were grown to ~80% confluency as described above and activated according to a previously published protocol by Yu et al. (2012). Astrocytes were activated with human TGF-β1 (Abcam, MA) for 24 h in serum-free DMEM/F12 containing no phenol red. Cells were then washed twice with 1× PBS to remove any residual TGF-β1. Treatments were then applied for 24 h (Table 1), after which cells and media were collected.

**Table 1:** List of treatments for experimental application.

Treatments for experimental application
• Hepatocyte growth factor-preconditioned neural stem/progenitorcell conditioned media **(HGF-NPC-CM)**
• Neural stem/progenitor cell conditioned media **(NPC-CM)**
• Recombinant human hepatocyte growth factor protein
• NSPC + HGF [20 ng/ml] applied concurrently

### Preparation of Astrocyte Conditioned Media and Cell Lysate

Following activation and subsequent treatment, astrocyte CM was collected, transferred to 15 ml conical tubes on ice, and centrifuged at 4°C for 10 min at 16,000 rpm. CM was subsequently filtered using a 0.44 μm syringe filter unit to remove any residual cells and protein quantification using the BCA assay was performed prior to further experimental assays.

Following activation and subsequent treatment, astrocytes were washed three times in ice-cold 1× PBS and gently collected using a cell scraper. The cell suspension was transferred to Eppendorf tubes and centrifuged in a 4°C microcentrifuge at 16,000 rpm for 10 min. Media-containing supernatant was carefully discarded and the cells were lysed using ice-cold radio-immunoprecipitation assay buffer (RIPA; Thermo Scientific) supplemented with protease inhibitors according to the manufacturer’s protocol. Samples were passed through a 25 gauge needle five times until sufficiently homogenized and then centrifuged at 14,000 rpm for 15 min. The supernatant from each sample was carefully removed ensuring that the underlying pellet was not disturbed, and then transferred to a new low-bind Eppendorf tube. Protein quantification using the BCA assay was also performed.

### Quantification of GFAP Production in Astrocyte Cell Lysate

Western blot: 15 μg cell lysate was mixed with 5× sample buffer and heated at 100°C in a water bath for 5 min, then centrifuged at 15,000 rpm for 10 min. After samples were cooled, equal amounts of protein were loaded into the wells of an 8% sodium dodecyl sulfate (SDS) acrylamide gel and subsequently run for approximately 2 h at 100 V. Protein from the gel was transferred to a nitrocellulose membrane after being run for 1 h at 100 V. The gels were then carefully removed and membranes were stained with Ponceau S solution to confirm protein transfer and assess total protein load. The membranes were blocked for 1 h at room temperature in a blocking solution containing 5% nonfat skim milk in 0.1% Tween20 phosphate- buffered saline (PBST). The membranes were incubated at 4°C overnight with the designated primary antibodies in blocking solution: rabbit anti-GFAP (1:1,000; Millipore) or mouse anti-αTubulin (1:200; Santa Cruz Biotechnology) in PBST containing 5% nonfat skim milk. Next, the membranes were washed three times in PBST (5 min each) and incubated for 1 h at room temperature with the corresponding HRP-conjugated secondary antibody in blocking solution: anti-rabbit IgG (1:10,000, Thermo Scientific) or anti-mouse IgG (1:10,000, Thermo Scientific). Pierce ECL Western Blotting Substrate (ThermoFisher Scientific) was used according to manufacturer’s protocol for signal development. The membranes were exposed to film and developed. Densitometric analysis was performed using ImageJ software (NIH). Experiments were done in triplicate with *n* = 3.

Immunocytochemistry: As described previously, astrocytes were grown on Matrigel-coated glass coverslips in a 24 well plate. The cells were then fixed in 4% phosphate-buffered paraformaldehyde for 10 min at room temperature. The cells were gently washed in 1× PBS three times for 5 min each. Next, cells were incubated for 30 min at room temperature in blocking solution containing 3% bovine serum albumin, 3% goat serum, and 0.1% Triton X 100 in 1× PBS. The cells were incubated at 4°C overnight with the designated primary antibody in blocking solution: rabbit anti-GFAP (1:500; Millipore). The cells were then washed three times in 1× PBS (5 min each) and incubated for 1 h at room temperature with the corresponding secondary antibody in blocking solution: goat anti-rabbit Alexa 488 (1:200; Thermo Scientific), and DAPI nuclear stain (1:1,000; Thermo Scientific). The cells were washed 3 times in 1× PBS and coverslips were carefully mounted to Superfrost Plus slides (Thermo Scientific) with Mowiol mounting medium (Sigma-Aldrich). Coverslips (*n* = 3 per sample) were imaged using an epifluorescence microscope (Zeiss Axioplan 2, Carl Zeiss, Toronto, ON, Canada) using standardized camera settings with three random fields per coverslip. Staining was quantified using a custom script in Image J and presented as the total area of staining per cell relative to TGFβ-activated astrocyte controls. 20× images were converted from 16-bit color to 8-bit grayscale. Noise removal was performed to remove fluorescent artifacts using the “Despeckle” function in ImageJ (removes specks of signal that are 1 px^2^ in area). A signal threshold of 15–255 grayscale was applied for staining detection. This was held constant across all images. An area fraction (% area) of staining was then calculated and converted to an actual area by taking the product of the area fraction to the total image area. To normalize for the total number of cells in each field of view, the “Find Maxima” function with a noise tolerance of 25 was used to find nuclei foci, and the number (in counts) was outputted. The final metric that was computed was expressed as positive staining area per cell in inches^2^.

### Quantification of CSPG Production in Astrocyte Conditioned Media

Slot blot: Following protein quantification, 15 μg of CM sample in 200 μl of double distilled water was loaded into the wells of a Bio-Dot SF microfiltration apparatus (Bio-Rad, Mississauga, Canada) and transferred to an underlying nitrocellulose membrane, following the manufacturer’s protocol. The membranes were then immunoprobed as described above for mouse anti-CSPG (C56S; 1:1,000; Sigma) and corresponding anti-mouse IgG (1:10,000, Thermo Scientific). The membranes were exposed to film and developed as described above. Densitometric analysis was performed using ImageJ software (NIH). Experiments were done in triplicate with *n* = 3.

Immunocytochemistry: Astrocytes were grown on Matrigel-coated glass coverslips in a 24-well plate, fixed, and prepared as previously described. Next, cells were incubated for 30 min at room temperature in blocking solution and incubated at 4°C overnight with the designated primary antibody in blocking solution: mouse anti-CSPG (C56S; 1:1,000; Sigma). The cells were then washed three times in 1× PBS (5 min each) and incubated for 1 h at room temperature with the corresponding secondary antibody in blocking solution: goat anti-mouse Alexa 568 (1:200; Thermo Scientific) and DAPI nuclear stain (1:1,000; Thermo Scientific). The cells were washed three times in 1× PBS and coverslips were carefully mounted to Superfrost Plus slides (Thermo Scientific) with Mowiol mounting medium (Sigma-Aldrich). Coverslips (*n* = 3 per sample) were imaged using an epifluorescence microscope (Zeiss Axioplan 2, Carl Zeiss, Toronto, ON, Canada) using standardized camera settings with three random fields per coverslip. Staining was quantified using a semi-automated ImageJ macros as previously described, and presented as the total area of staining per cell relative to TGFβ-activated astrocyte controls.

### Neurite Outgrowth Assay

#### Maintenance and Expansion of hiPSC Line

The human induced pluripotent stem cell (hiPSC) line, BC1, derived from human adult bone marrow hematopoietic cells, was adapted to serum and feeder-free conditions using E8 medium (Essential 8 medium, Life Technologies, CA, USA) and expanded on Matrigel in mTeSR1.

#### Generation of NPCs From hiPSCs

Dual-SMAD inhibition using SB431542 and Noggin in monolayer was used to differentiate hiPSCs to NPC, as previously described by Chambers et al. (2009). A monoclonal line of NPCs was established by two cycles of single cell-neurosphere formation in Ultra-Low Adhesion Plates (Corning, Tewksbury, MA, USA) with initial plating at 10,000 cells/ml. NPCs were maintained in NPC expansion media consisting of DMEM/F12 + Glutamax (Gibco) + B27 Supplement without Vitamin A (Gibco) + bFGF (20 ng/ml; Peprotech) + EGF (20 ng/ml; Peprotech) + 1% Penicillin/Streptomycin (Invitrogen).

#### HiPSC-NPC Neuronal Differentiation

HiPSC-NPCs were plated onto Poly-L-Ornithine/laminin-coated dishes for further differentiation to neurons using neuron differentiation media (NDM) as described by Palm et al. (2015), consisting of neurobasal media (Gibco) + N2 Supplement (Gibco) + B27 Supplement (Gibco) + BDNF (BDNF; 10 ng/ml; Peprotech) + (GDNF; 10 ng/ml; Peprotech) + TGF-β3 (1 ng/ml; Peprotech) + cyclic adenosine monophosphate (cAMP; 500 μM; Sigma Aldrich). NDM was changed every 2 days for 6 weeks prior to harvesting of neurons.

#### Reactive Astrocyte-Neuron Co-culture

5 × 10^4^ astrocytes per well were plated onto Matrigel-coated 12 mm glass coverslips in a 24-well plate. Once cells were grown to confluency, they were activated and treated as previously described. Following treatment, residual media was removed and 1 × 10^4^ hiPSC-NPC-derived neurons/well were plated onto the confluent monolayer of astrocytes and co-cultured for 72 h.

#### Immunofluorescence Staining

Neurons and astrocytes were labeled by immunofluorescence staining. The cells were fixed in 4% phosphate-buffered paraformaldehyde for 10 min at room temperature and gently washed in 1× PBS three times for 5 min each. Next, cells were incubated for 1 h at room temperature in a blocking solution containing 5% bovine serum albumin, 3% goat serum, and 0.1% Triton X 100 in 1× PBS. The cells were then incubated at 4°C overnight with the designated primary antibodies in blocking solution: mouse anti-MAP2 (1:500; Millipore) or rabbit anti-GFAP (1:500; Millipore), to label neurons and astrocytes, respectively. The cells were then washed three times in 1× PBS (5 min each) and incubated for 1 h at room temperature with the corresponding secondary antibody in blocking solution: goat anti-mouse Alexa 488 (1:500; Thermo Scientific) or goat anti-rabbit Alexa 568 (1:500; Thermo Scientific), and DAPI nuclear stain (1:1,000; Thermo Scientific). The cells were washed three times in 1× PBS and coverslips were carefully mounted to Superfrost Plus slides (Thermo Scientific) with Mowiol mounting medium (Sigma-Aldrich). Coverslips (*n* = 3 per sample) were imaged at 20× magnification using a Nikon Eclipse Ti microscope using standardized camera settings with four randomly selected fields per coverslip. Quantification of total neurite length per cell was performed using the NeuronJ plug-in available in ImageJ software (NIH).

#### Statistical Analysis

GraphPad Prism (La Jolla, CA, USA) was utilized for all statistical analyses. Quantitative data were expressed as the ± standard error of the mean (SEM) with differences among groups assessed by one-way ANOVA with Tukey’s *post hoc* test. Statistical significance was set at *p* ≤ 0.05.

## Results

### HGF Enhances NPC Viability During Oxidative Stress Exposure

Oxidative stress is a well-known pathogenic process in CNS injury resulting in cellular apoptosis (Chan, 2001; Shytle et al., 2007). Here, we utilized hydrogen peroxide to induce oxidative stress in adult rat spinal cord-derived NPCs and investigated the effect of concurrent HGF treatment on cell viability. The MTT assay was utilized to measure cell viability following oxidative stress exposure.

Exposure of NPCs to 500 μM of H_2_O_2_ for 24 h significantly decreased the viability of these cells, a finding that is congruent with previous studies utilizing the same cell type and source (Hachem et al., 2015). Relative to untreated NPCs, H_2_O_2_-treated cells demonstrated approximately 40% cell viability (85,009 ± 3,290 vs. 137,111 ± 1,920 cells; *p* < 0.0001; Figure 1B) confirming the utility of this model as an apoptosis-inducing environment *in vitro*.

We further investigated whether concurrent treatment of NPCs with HGF protein conferred protection against H_2_O_2_-induced apoptosis. NPCs exposed to H_2_O_2_ were also treated with varying concentrations of HGF (10, 20, or 50 ng/ml) for 24 h (Hu et al., 2010). HGF treatment at concentrations of 20 and 50 ng/ml demonstrated the most notable effects on cell viability relative to H_2_O_2_-treated NPC controls (115,447 ± 3,839 cells, *p* < 0.0041; and 108,592 ± 1,097 cells, *p* < 0.0020; respectively). Based on these findings, a concentration of 20 ng/ml was used for future preconditioning and concurrent experimental HGF application.

### HGF-Preconditioned NPCs Demonstrate Reduced VEGF and Increased TIMP-1 Production

To characterize the secretory cytokine profile of HGF-preconditioned NPCs, proteomic analysis was conducted on CM using Rat Cytokine Array/Chemokine Array 27 Plex and Rat Vascular Injury P1-4-Plex kits (Eve Technologies). Out of the 31 cytokines that were evaluated, notable differences were observed in the levels of TIMP-1 and VEGF (Figure 1C). HGF-preconditioned NPCs demonstrated a notable increase in TIMP-1 production relative to untreated NPC-CM controls (3,034.52 ± 1,686.59 vs. 703.85 ± 205.84; *p* = 0.009). While both groups demonstrated a marked detection of VEGF levels relative to the other cytokines, HGF-preconditioned NPCs secreted less VEGF in comparison to untreated NPC controls (5,361.20 ± 836.88 vs. 7,773.04 ± 1,446.81 pg/ml; *p* < 0.0001).

### Combinatorial Treatment of Reactive Astrocytes With HGF-Enhanced NPC-CM Demonstrates Reduced GFAP Expression

**Figure 2:**
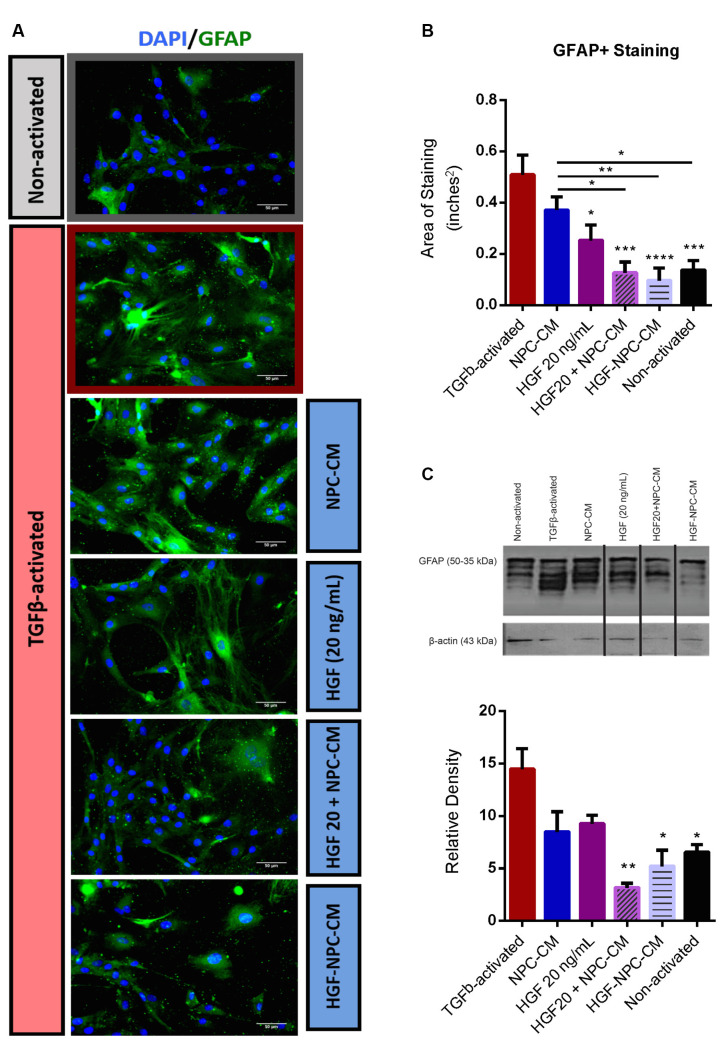
Combinatorial treatment of reactive astrocytes with HGF-enhanced NPC-CM demonstrates reduced glial fibrillary acidic protein (GFAP) expression. **(A,B)** Immunostaining of non-activated and TGFβ-induced reactive astrocytes with reactivity marker GFAP (green) and DAPI (blue). TGFβ-activated astrocytes demonstrate a significant increase in GFAP expression relative to non-activated astrocytes. Subsequent administration of HGF alone as well as combinatorial treatment (preconditioned and concurrently applied HGF and NPC-CM) demonstrated a significant reduction in GFAP expression relative to TGFβ-activated controls. Combinatorial treatment also proved more effective than NPC-CM alone. Data are expressed as mean ± SEM; one-way ANOVA with Tukey’s *post hoc* test; *p ≤ 0.5, **p ≤ 0.01, ***p ≤ 0.001, ****p ≤ 0.0001. **(C)** Western blot of cell lysate probed with GFAP (bands ranging from 35 to 55 kDa) and β-actin loading control (43 kDa). TGFβ-activated astrocytes demonstrate a significant increase in GFAP expression relative to non-activated astrocytes. Only combinatory treatments (preconditioned and concurrently applied) demonstrated a significant reduction in GFAP production relative to TGFβ-activated controls. Data are expressed as mean ± SEM; one-way ANOVA statistical analysis with Tukey’s *post hoc* test was performed; *p ≤ 0.5, **p ≤ 0.01, ***p ≤ 0.001.

Upregulation of the intermediate filament protein GFAP, constitutes a characteristic marker of astrocyte reactivity (Ribotta et al., 2004; Yu et al., 2012). Area staining analysis in non-reactive astrocytes demonstrated significantly less GFAP relative to TGFβ1-activated astrocyte controls (0.14 ± 0.03 vs. 0.51 ± 0.07; *p* = 0.0002; Figures 2A,B), confirming the utility of this *in vitro* astrocyte activation protocol. Subsequent treatment of reactive astrocytes with HGF alone demonstrated a significant reduction in GFAP expression (0.26 ± 0.05; *p* = 0.0187), while treatment with NPC-CM alone yielded no significant decrease (0.38 ± 0.05; *p* = 0.4733). Combinatorial treatment, however, whether *via* concurrent (0.12 ± 0.04) or HGF-preconditioned NPC-CM application (0.09 ± 0.05), demonstrated the most substantial reductions in GFAP+ staining, relative to TGFβ1-activated astrocyte controls (*p* < 0.0001 and *p* < 0.0001, respectively; Figures 2A,B). Combinatorial treatment (concurrent and preconditioned) of reactive astrocytes also demonstrated GFAP levels comparable to that of unstressed, non-activated astrocytes (0.14 ± 0.03; Figures 2A,B). Statistical analyses of the total cell count between untreated astrocyte and HGF-NPC-CM treated groups were performed, with an increase observed in the latter (*p* = 0.02; Supplementary Figure 1).

Akin to immunostaining, Western blot densitometric analysis in non-reactive astrocytes demonstrated significantly less GFAP production relative to TGFβ1-activated astrocyte controls (6.60 ± 0.67 vs. 14.57 ± 1.85; *p* = 0.0139; Figure 2C), confirming the utility of this activation method. Concurrent administration of HGF and NPC-CM (3.16 ± 0.38, *p* = 0.0008) as well as HGF-preconditioned NPC-CM (5.23 ± 1.50, *p* = 0.0044) yielded a significant reduction in GFAP relative to TGFβ1-activated controls (Figure 2C). Combinatorial treatment (concurrent and preconditioned) of reactive astrocytes also demonstrated GFAP levels comparable to that of unstressed, non-activated astrocytes (6.60 ± 0.67; *p* = 0.5248 and *p* = 0.9793, respectively; Figure 2C).

### Combinatorial Treatment of Reactive Astrocytes With HGF-Enhanced NPC-CM Demonstrates Reduced CSPG Deposition

**Figure 3:**
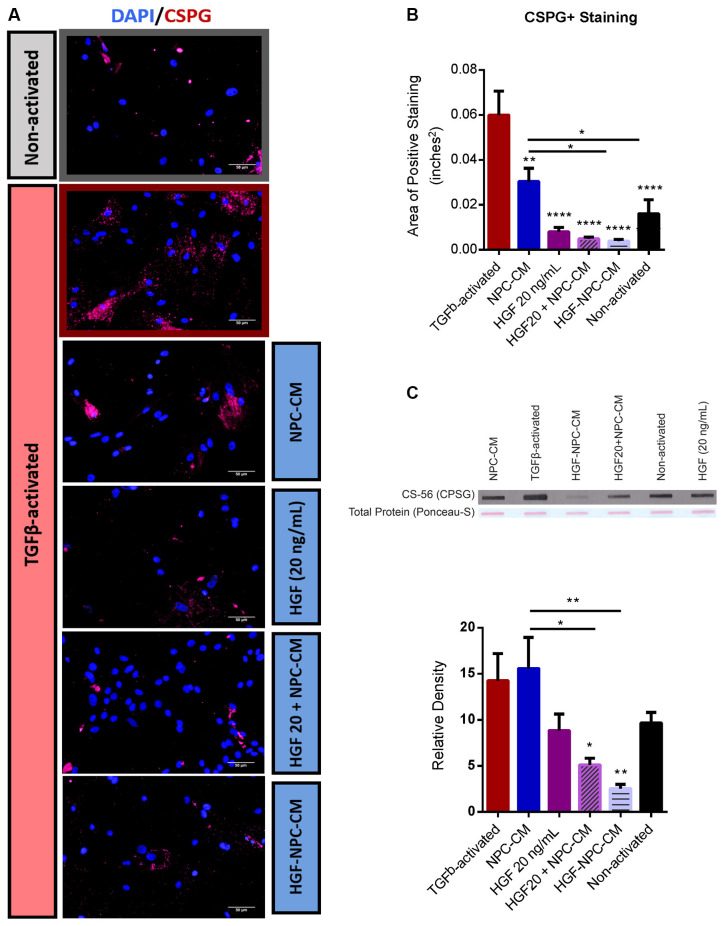
Combinatorial treatment of reactive astrocytes with HGF-enhanced NPC-CM demonstrates reduced CSPG deposition. **(A,B)** Immunostaining of non-activated and TGFβ-induced reactive astrocytes with reactivity marker CSPG (green) and DAPI (blue). TGFβ-activated astrocytes demonstrate a significant increase in CSPG expression relative to non-activated astrocytes. All treatment groups demonstrated a significant reduction in CSPG expression relative to TGFβ-activated astrocyte controls; Combinatorial treatment (preconditioned and concurrently applied) proved more effective than NPC-CM alone. Data are expressed as mean ± SEM; One-way ANOVA statistical analysis with Tukey’s *post hoc* test; *p ≤ 0.05, **p ≤ 0.01, ****p ≤ 0.0001.** (C)** Slot blot analysis of astrocyte CM probed with CSPG. While treatment of TGFβ-activated astrocytes with NPC-CM and HGF alone revealed trending reductions in CSPG expression, only combinatorial treatment (preconditioned and concurrently applied) demonstrated significant reductions. Combinatorial treatment also proved more effective than NPC-CM alone. Data are expressed as mean ± SEM and normalized to ponceau total protein load. One-way ANOVA statistical analysis with Tukey’s *post hoc* test was performed; *p ≤ 0.05, **p ≤ 0.01.

Marked upregulation and deposition of CSPGs is a key hallmark of reactive astrogliosis and a major target for therapeutic intervention in CNS injury (Morgenstern et al., 2002; Fitch and Silver, 2008; Yu et al., 2012). Area staining analysis of non-reactive astrocytes demonstrated significantly less CSPG relative to TGFβ1-activated astrocyte controls (0.016 ± 0.006 vs. 0.06 ± 0.01; *p* < 0.0001; Figures 3A,B), confirming the utility of this *in vitro* astrocyte activation protocol. Concurrent (HGF + NPC-CM) treatment (0.005 ± 0.001) and HGF-preconditioned NPC-CM (0.004 ± 0.001) demonstrated notable reductions in CSPG+ staining relative to NPC-CM treatment alone (*p* = 0.0290 and *p* = 0.0205, respectively; Figures 3A,B). Combinatorial treatment (concurrent and preconditioned) of reactive astrocytes also demonstrated CSPG levels comparable to that of unstressed, non-activated astrocytes (0.016 ± 0.006; *p* = 0.7286 and *p* = 0.6480, respectively; Figures 3A,B).

In the slot blot assay, concurrent administration of HGF and NPC-CM (5.18 ± 0.69) as well as HGF-preconditioned NPC-CM (2.56 ± 0.39) yielded a significant reduction in CSPG deposition relative to TGF β1-activated controls (14.37 ± 2.86; *p* = 0.0405 and *p* = 0.0045, respectively; Figure 3C). Combinatorial concurrent (HGF + NPC-CM) treatment and HGF-preconditioned NPC-CM also demonstrated notable reductions relative to NPC-CM treatment alone (15.65 ± 3.33; *p* = 0.0135 and *p* = 0.0013, respectively; Figure 3C). Combinatorial treatment (concurrent and preconditioned) of reactive astrocytes also demonstrated CSPG levels that were not significantly different than that of unstressed, non-activated astrocytes (9.73 ± 1.07; *p* = 0.6248 and *p* = 0.1715, respectively; Figure 3C). Of note, contrary to the stain, NPC-CM alone did not reduce the total CSPG on the slot blot.

### Combinatorial Treatment of Reactive Astrocyte-Neuron Co-cultures Demonstrates Increased Neurite Outgrowth of hiPSC-Neurons

A key consequence of astrocyte reactivity and glial scarring is inhibition of neurite outgrowth, the effect of which is largely mediated by the production of CSPGs and other inhibitory ECM molecules (McKeon et al., 1991; Dou and Levine, 1994; Mckeon et al., 1995, 1999; Yiu and He, 2006). Here, we mimic a growth-inhibitory environment through TGF-β1-induced astrocyte reactivity, which results in increased CSPG deposition (Figures 3A–C). HiPSC-derived neurons were co-cultured atop this feeder layer of reactive astrocytes and singular or combinatorial treatments were then administered. After 4 days, the total mean neurite length per cell was assessed.

**Figure 4:**
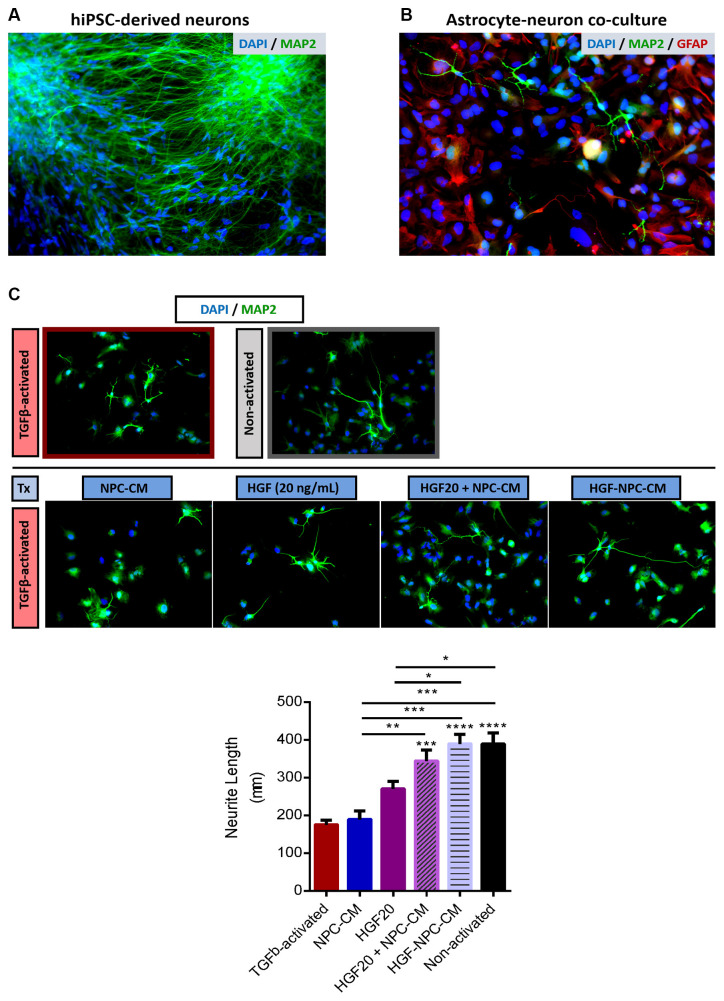
Combinatorial treatment with HGF-enhanced NPC-CM demonstrates increased neurite outgrowth of hiPSC-neurons in reactive astrocyte co-culture. **(A)** Immunostaining of hiPSC-derived neurons with neuronal and dendritic marker MAP2 (green) and DAPI (blue). Representative image taken at 20×. **(B)** hiPSC-derived neurons were plated onto a confluent monolayer of TGFβ-activated astrocytes. Immunostaining of neuronal and dendritic marker MAP2 (green), astrocyte reactivity marker GFAP (red), and DAPI (blue). **(C)** Treatments were applied to reactive astrocyte-neuron co-culture for a span of 4 days and imaged at 20× thereafter. There was approximately a 50% decrease in the total neurite length of neurons plated atop reactive astrocyte substrates relative to non-activated astrocytes. Combinatorial treatment applied to reactive-neuron co-cultures demonstrated a significant increase in neurite length compared to reactive astrocyte controls; the extent of this growth was comparable to that of the non-activated astrocyte substrate. Combinatorial treatment also proved more effective than either HGF or NPC-CM alone. Data are expressed as mean ± SEM; one-way ANOVA statistical analysis with Tukey’s *post hoc* test; *p ≤ 0.05, **p ≤ 0.01, ***p ≤ 0.001, ****p ≤ 0.0001.

HiPSC-derived neurons are indicated by the neuronal and dendritic marker MAP2 (Figure 4A), while reactive astrocytes and hiPSC-derived neurons are indicated in co-culture *via* GFAP and MAP2 staining, respectively (Figure 4B).

TGFβ1-activated astrocytes demonstrated a significant reduction in neurite length, approximately two times less than that of non-activated astrocytes (177.26 ± 11.98 μm; *p* < 0.0001; Figure 4C), confirming the utility of this activation protocol as inhibitory to neurite outgrowth.

Treatment of co-cultures with NPC-CM or HGF protein alone did not demonstrate statistically significant differences in neurite length relative to reactive astrocyte-neuron controls, although the data is indicative of trending reductions (192.36 ± 18.29 μm; *p* = 0.9974 and 274.24 ± 15.1 μm; *p* = 0.0912, respectively; Figures 4B,C). Co-cultures that were subjected to concurrent administration of HGF and NPC-CM and HGF-preconditioned NPC-CM demonstrated a significant increase in total mean neurite length relative to TGFβ1-activated controls (347.37 ± 25.44 μm; *p* = 0.0010 and 392.68 ± 23.85 μm; *p* < 0.0001, respectively; Figure 4C). Interestingly, combinatorial treatments, both concurrent and preconditioned treatments demonstrated an increase in neurite length comparable to that of non-activated co-cultures (394.18 ± 27.03 μm), respectively.

Combinatorial concurrent (HGF + NPC-CM) treatment and HGF-preconditioned NPC-CM demonstrated notable increases in neurite length relative to NPC-CM treatment alone (*p* = 0.0001, respectively; Figure 4C). HGF preconditioned NPC-CM also proved more effective in promoting neurite outgrowth relative to HGF treatment alone (*p* = 0.0228; Figure 4C).

## Discussion

Stem-cell-based therapies are a promising and transformative approach for addressing the dynamic and multifactorial nature of secondary injury following CNS trauma. NPCs are particularly attractive for transplantation given their cell replacement capacity and trophic support (Hawryluk et al., 2012b; Mothe and Tator, 2013). Reactive astrogliosis and formation of the glial scar ultimately present a substantial physical and chemical barrier to regeneration (Silver and Miller, 2004). While the majority of therapeutics have been focused on reducing neuroinflammation through drug-based interventions (Bye et al., 2007; Guimarães et al., 2010; Lopes et al., 2016), therapeutic interventions have expanded to strategically enhance and utilize the reparative potential of NPC secreted factors as a means of modifying the post-injury glial environment to one more conducive for regeneration (Liang et al., 2014; Doeppner et al., 2017; Merianda et al., 2017).

HGF has been investigated as a therapeutic agent alone as well as in combination with cellular therapies for CNS injury and neurodegenerative disease, most notably for its neurotrophic and regenerative capacity (Hamanoue et al., 1996; Hu et al., 2010; Wong et al., 2014). To our knowledge, this is the first study to assess the efficacy of HGF as a preconditioning and combinatorial agent in boosting the secretory and trophic capacity of NPCs to attenuate astrocyte reactivity. Here, we demonstrate that HGF preconditioning induces distinct changes in secretory cytokine levels. Moreover, HGF-enhanced NPC-CM effectively attenuates inhibitory hallmarks of astrocyte reactivity while promoting concurrent neurite outgrowth to a greater extent than either treatment option alone.

### Combinatorial Treatment With HGF-Enhanced NPC-CM Attenuates Astrocyte Reactivity

In the current study, we report a significant reduction in astrocyte reactivity following combinatorial treatment (via HGF-preconditioned NPC-CM or concurrent administration at high concentrations of HGF). Combinatorial treatment (concurrent and preconditioned) of reactive astrocytes also demonstrated GFAP and CSPG levels comparable to that of unstressed, non-activated astrocytes. This might indicate that the proposed treatment could aid in restoring CSPG and GFAP levels to that of reactive astrocyte baseline levels after injury.

The regenerative potential of HGF has been highlighted in the development, maintenance, and repair of tissue; moreover, HGF has been described to reduce glial scarring in the injured CNS (Tönges et al., 2011; Jeong et al., 2012; Matsumoto et al., 2014; Wong et al., 2014). In a model of middle cerebral artery occlusion, HGF administration alone not only reduced glial scar tissue and thickness but promoted synaptogenesis and angiogenesis (Shang et al., 2011). Several studies have noted the localized upregulation of HGF and its c-Met receptor predominately in reactive astrocytes at and around the injury site (Nagayama et al., 2004; Shimamura et al., 2007). Moreover, this upregulated expression was not associated with astrocytic proliferation or migration, which may further be indicative of a reparative response mechanism to the inflammatory and inhibitory processes induced by reactive astrocytes in the glial scar (Nagayama et al., 2004; Shimamura et al., 2007).

Our lab, among others, has demonstrated reductions in reactive astrogliosis and glial scarring following NPC transplantation; in the injured spinal cord, NPC transplantation yielded reductions in astrogliosis and glial scarring as measured by GFAP and CSPG (Wilcox et al., 2014). Similarly, in TBI, NPC transplantation significantly reduced astrogliosis in the corpus callosum (Sullivan and Armstrong, 2017). While the mechanisms of these effects are not fully understood, they attest to the reparative but perhaps limited, glial-modifying potential of NPC-secreted factors. In the injured spinal cord, HGF-overexpressing mesenchymal stromal cells markedly reduced the extent of astrocyte activation and CSPG deposition around the lesion site while increasing axonal growth beyond the glial scar (Jeong et al., 2012).

### Combinatorial Treatment With HGF-Enhanced NPC-CM Promotes Neurite Outgrowth

HiPSCs are a clinically attractive source for cell replacement therapies and circumvent a number of ethical constraints arising from embryonic stem cell sources (Angelos and Kaufman, 2015). While primary rodent cultures and neuronal cell lines have been widely employed in *in vitro* studies, they may not fully model human biology. The utilization of hiPSC-derived neurons may help overcome this challenge and serve as an amenable source for neurite outgrowth assays (Druwe et al., 2016). The capacity of neurons to project membrane extensions from their cell bodies is indicative of cell function and health, and as such, we measured mean total neurite length post-treatment (Yamamoto et al., 2012). In the current study, combinatorial treatment of reactive astrocytes with HGF-enhanced NPC-CM (via preconditioning or concurrent administration) resulted in greater neurite outgrowth of hiPSC-neurons compared to either treatment option alone.

In maturing sympathetic neurons, HGF has been shown to promote survival and growth *via* PI3 kinase and MAP kinase-dependent mechanisms (Thompson et al., 2004). Following optic nerve injury, HGF administration has also been shown to promote long-term survival and axonal regeneration of retinal ganglion cells (Wong et al., 2014). Furthermore, in a model of cerebral infarction, HGF gene transfer resulted in enhanced neurite extension and functional recovery (Shimamura et al., 2006).

While we did not observe any significant increases in neurite outgrowth following NPC-CM treatment, numerous studies have demonstrated an upregulation of neurotrophic factors such as BDNF, CNTF, GDNF, and NGF, all of which may mediate attenuation of glial scar formation and axonal growth (Lu et al., 2003; Chu et al., 2004; Ziv et al., 2006). In a model of ischaemic stroke, transplanted NPCs not only secreted trophic factors such as VEGF, but induced host expression of guidance molecules that regulated dendritic sprouting, axonal plasticity, and axonal transport (Andres et al., 2011). More recently, the growth-promoting effect of NPC-conditioned medium has also been observed in injury-conditioned neurons (Merianda et al., 2017). This growth was linked to enhanced transcription and localization of growth-associated genes in growing axons (Merianda et al., 2017).

In the current study, whether the observed increase in neurite outgrowth following combinatorial treatment was attributed to direct or indirect actions on neuritogenesis or neuron-glia interactions, respectively, remains unclear. However, given the significant reduction in astrocytic CSPG production following combinatorial treatment, it may be reasonable to attribute the observed neurite outgrowth to a lack of this regenerative constraint.

### HGF-Preconditioned NPCs Demonstrate Increased TIMP-1 and Reduced VEGF Production

Of the cytokines that we assessed in the current study, TIMP-1 was notably increased in HGF-preconditioned NPCs. Although multiple mechanisms are likely implicated in the regulation of astrocyte reactivity observed in the current study, upregulation of the endogenous matrix metalloproteinase (MMP) inhibitor, TIMP-1, may serve as one such mechanism. A study by Hsu et al implicated MMP-9 as integral to glial scar formation and cytoskeleton-mediated astrocyte migration (Hsu et al., 2008). Moreover, in the injured spinal cord, MMP-9-null mice demonstrated less severe glial scarring and CSPG expression than uninjured controls (Hsu et al., 2008). Upregulation of the endogenous MMP-9 inhibitor in the current study, may be one such explanation for the notable attenuation in GFAP and CSPG expression we observed following treatment.

We also observed a significant decrease in VEGF production in HGF-preconditioned NPCs relative to untreated controls. Increased VEGF expression has been reported in the injured spinal cord, particularly in activated astrocytes and microglia around the injury site (Nesic et al., 2010). Similarly, in TBI, VEGF upregulation has been observed and localized predominately to reactive astrocytes, facilitating neoangiogenesis in correlation with reactive astrogliosis (Salhia et al., 2000). Interestingly the regulatory role of HGF in augmenting VEGF-driven endothelial angiogenesis has recently been investigated (Xin et al., 2001; Sulpice et al., 2009). Studies have highlighted the particularly synergistic effect of HGF and VEGF on enhancing endothelial cell survival and neovascularization post-injury through upregulation of the pro-survival genes Bcl-2 and A1 and enhancing ERK1/2 and p38 kinase signaling, respectively (Xin et al., 2001; Sulpice et al., 2009). Furthermore, in ischemic skeletal muscle, co-transfer of VEGF and HGF genes rendered robust angiogenic effects (Makarevich et al., 2012). In the context of CNS injury, this secondary effect may serve as yet another mechanism for tissue repair, attesting to the multifaceted therapeutic efficacy of HGF-enhanced NPC-CM.

Characterization of cellular CM in our current study provides important insight into key cytokines that are modified in NPCs following HGF pre-treatment.

### Technical Considerations and Future Directions

*In vitro* preconditioning offers an inexpensive and non-viral approach for modulating cellular secretory capacity, eliciting a global response to a distinct stimulus (Lu et al., 2003; Hu et al., 2009). Cellular preconditioning also has the advantage of bypassing inflammatory issues associated with implanted pumps or invasive catheters designed for co-administration of growth factors, as well as mutational and neoplasmic risks associated with genetic manipulation (Lu et al., 2003; Hu et al., 2009).

While *in vitro* systems are particularly advantageous in their ability to model injury and assess treatment efficacy, culture conditions, cell origin, and heterogeneity are all factors that can limit clinical translation (Lu et al., 2003; Hawryluk et al., 2012a). Our work, which involves medium derived from preconditioned adult rat-NPCs, is susceptible to these limitations. In order to maintain cell identity, NPCs were grown in cell-specific medium and passage-matched. Moreover, it is important to consider the impact of environmental cues on trophin expression, as cell behavior after transplantation is subject to the heterogeneous and dynamic milieu of the injured CNS (Hawryluk et al., 2012b).

We assessed neurite outgrowth of hiPSC-neurons in a rodent astrocyte co-culture system. Other studies have indeed utilized co-culture systems with human neurons and rodent astrocytes, demonstrating improved functional neuronal maturation (Johnson et al., 2007; Tang et al., 2013; Odawara et al., 2014). However, rodent astrocytes differ significantly from human astrocytes (Oberheim et al., 2009; Zhang et al., 2016). As such, fully human-derived models hold considerable value in effectively studying CNS injury and assessing functional interactions following treatment. Nonetheless, our findings provide important insight into cellular interactions following treatment and are a promising and translationally relevant first step in informing fully human *in vitro* models. While we assessed mean total neurite length in the current study, it would also be informative to confirm polarity establishment as it relates to axon growth using either Tau-1 immunostaining or live-cell imaging of kinesin-1, an early axonal marker (Yamamoto et al., 2012). Future studies should also assess differences in dendritic branching and number between groups. Moreover, while the number of plated neurons was kept consistent, cell survival should also be assessed both with and without treatment.

Whether the changes in TIMP-1 and VEGF production after HGF preconditioning mediate regulatory effects on astrocyte reactivity and to what extent, warrants further mechanistic investigation, and such should be the focus of future studies. Furthermore, the secretory cytokine profile of preconditioned NPCs in the current study may likely reflect cytokine production at the time of transplant. Future *in vivo* work is necessary to confirm changes in trophin expression after engraftment.

Our findings are largely informative for future pre-clinical studies as well as dosing and administration needs. The *in vitro* model used in the current study serves as a necessary first step in optimizing our HGF preconditioning strategy, assessing changes in cytokine production, and identifying potential factors involved in astroglial modification and neurite outgrowth.

## Conclusion

This is the first study to profile and investigate the astroglial-modifying efficacy of HGF-preconditioned NPCs. We show that the application of a non-viral, NPC preconditioning treatment paradigm can effectively attenuate inhibitory hallmarks of reactive astrogliosis and promote neurite outgrowth to a greater extent than either treatment alone. These effects may be mediated in part by distinct changes in NPC secretory cytokine levels. Harnessing or enhancing the reparative secretory potential of NPCs *via* either HGF preconditioning or concurrent administration may serve as a suitable therapy for modifying the inhospitable injury milieu to one more permissive for repair and regeneration. Combinatorial cellular and growth factor therapies that collectively encompass cell replacement alongside mitigation of inhibitory astroglial components hold much promise for neuroregeneration and functional recovery in the injured CNS. Overall, these findings have important implications for the optimization of current cell-based strategies for CNS injury and neurodegenerative disease. Taken together, these data warrant further mechanistic and pre-clinical investigation into the reparative potential of HGF-enhanced NPCs as a potentially clinically translatable therapy.

## Data Availability Statement

The original contributions presented in the study are included in the article/Supplementary Material, further inquiries can be directed to the corresponding author.

## Author Contributions

JH: data collection and assembly, analysis, manuscript editing, analysis and interpretation, and manuscript writing. RD: study inception and experimental design, data collection and assembly, analysis and interpretation, and manuscript writing. MK: study inception and experimental design, data collection. CA: data collection and assembly and manuscript editing. MF: study inception and experimental design, data interpretation, manuscript editing and approval. All authors contributed to the article and approved the submitted version.

## Conflict of Interest

The authors declare that the research was conducted in the absence of any commercial or financial relationships that could be construed as a potential conflict of interest.

## Publisher’s Note

All claims expressed in this article are solely those of the authors and do not necessarily represent those of their affiliated organizations, or those of the publisher, the editors and the reviewers. Any product that may be evaluated in this article, or claim that may be made by its manufacturer, is not guaranteed or endorsed by the publisher.

## Footnotes

^1^ https://www.evetechnologies.com

^2^ https://software.broadinstitute.org/morpheus/

## Supplementary Materials

Supplementary Figure 1 The average total DAPI+ cell per field-of-view was evaluated for each replicate (*n* = 3); GFAP and CSPG were co-labeled. HGF-NPC-CMs contained a greater number of cells per field-of-view than that of untreated cells (*p* = 0.02). Error bars represent ± SEM.
