# Supplementary figures and images for: Hepatocyte Growth Factor-Preconditioned Neural Progenitor Cells Attenuate Astrocyte Reactivity and Promote Neurite Outgrowth
Source: Front Cell Neurosci. 2021 Dec 9;15:741681. doi: 10.3389/fncel.2021.741681 (PMC8695970; doi:10.3389/fncel.2021.741681)

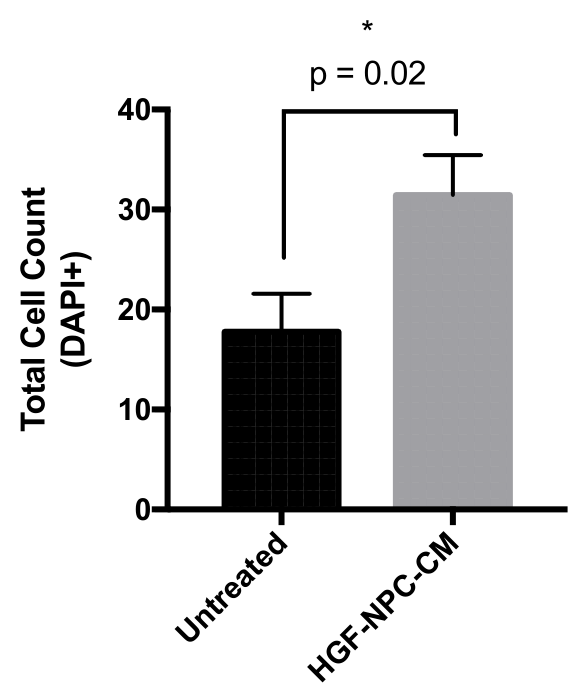

Supplement: Supplementary Figure 1 — The average total DAPI+ cell per field-of-view was evaluated for each replicate (n = 3); GFAP and CSPG were co-labeled. HGF-NPC-CMs contained a greater number of cells per field-of-view than that of untreated cells (p = 0.02). Error bars represent ± SEM. [file Image_1.TIFF]
